# Coronary complexity and inflammatory burden in HIV-infected patients with acute coronary syndrome: a two-year prospective cohort study in China

**DOI:** 10.3389/fcvm.2025.1702174

**Published:** 2026-01-19

**Authors:** Qiang Wu, Liqin Sun, Chunxiao Lu, Lina Zhang, Siwei Jiang, Wenhui Xu, Niuniu Li, Yifan Zhang, Meixia Chen, Xianjia Ning, Jinghua Wang, Fei Wang, Juan Meng, Hong Gao

**Affiliations:** 1 Department of Geriatrics, National Clinical Research Center for Infectious Diseases, Shenzhen Third People's Hospital, the Second Hospital Affiliated of Southern University of Science and Technology, Shenzhen, China; 2 Department of Infectious Diseases, National Clinical Research Center for Infectious Diseases, Shenzhen Third People's Hospital, the Second Hospital Affiliated of Southern University of Science and Technology, Shenzhen, China; 3 Department of Pediatrics, National Clinical Research Center for Infectious Diseases, Shenzhen Third People's Hospital, the Second Hospital Affiliated of Southern University of Science and Technology, Shenzhen, China; 4 Department of Liver Diseases, Shenzhen Third People’s Hospital, Shenzhen, China; 5 Medical Affairs Department, Shenzhen Third People’s Hospital, Shenzhen, China; 6 Center of Clinical Epidemiology, Shenzhen Third People's Hospital, the Second Hospital Affiliated of Southern University of Science and Technology, Shenzhen, China

**Keywords:** acute coronary syndrome, coronary angiography, HIV infection, inflammation, triglycerides

## Abstract

**Background and objectives:**

People living with HIV (PLWH) are increasingly at risk for cardiovascular disease (CVD) due to chronic inflammation, metabolic dysregulation, and long-term antiretroviral therapy (ART). However, limited data exist regarding coronary anatomy and long-term outcomes in PLWH with acute coronary syndrome (ACS), particularly in Chinese populations. This study aimed to compare coronary angiographic characteristics, metabolic-inflammatory profiles, and 2-year prognosis between PLWH and HIV-uninfected patients with ACS.

**Methods:**

We conducted a single-center observational cohort study including 129 patients with a first episode of ACS between 2019 and 2023. 43 PLWH were analyzed with 86 HIV-uninfected controls. Coronary artery lesions were assessed by angiography and SYNTAX score. Laboratory parameters including CRP and lipid levels, among others, were collected. The primary outcome was the incidence of major adverse cardiovascular and cerebrovascular events during the 2-year follow-up period.

**Results:**

PLWH had significantly higher SYNTAX scores compared to HIV-free patients (21.56 vs. 16.77, *P* = 0.001). They also showed elevated levels of LDL-C (3.18 vs. 2.66 mmol/L, *P* = 0.009) and CRP (median 9.10 vs. 5.10 mg/L, *P* < 0.001). At 2 years, PLWH had a higher rate of ACS recurrence (18.6% vs. 7.0%, *P* = 0.089), but mortality and other adverse events were similar. In multivariate analyses, HIV status was not an independent predictor of relapse, while calcified lesions were significantly associated with relapse.

**Conclusion:**

PLWH with ACS have more complex coronary heart disease and a higher risk of recurrence, partly due to vascular calcific changes. These findings suggest the need for enhanced metabolic monitoring and individualized secondary prevention strategies in this high-risk population.

## Introduction

Acute coronary syndrome (ACS) is a clinical syndrome characterized by myocardial ischemia and infarction caused by insufficient coronary blood supply. ACS is typically the first clinical manifestation of cardiovascular disease (CVD) and is a major cardiovascular event. The 2023 ESC Guidelines for the Management of ACS reported an estimated 5.8 million new cases of ischemic heart disease in 57 member states (1). Ischemic heart disease is the deadliest form of CVD, accounting for 38% of CVD deaths in women and 44% in men (2). An estimated 33.1 to 45.7 million people worldwide live with HIV (PLWH) as of 2022, with 1.3 million new infections projected for the year (3). Over 40.4 million people have died from AIDS-related diseases since the beginning of the pandemic (3). A recent modeling study predicted that 78% of PLWH will develop CVD by 2030 (4).

This correlation between HIV infection and CVD has garnered increasing attention. While the deleterious effects of HIV on the immune system have been well studied, the pathological mechanisms underlying coronary disease remain unclear and likely involve a combination of direct viral effects, side effects of antiretroviral therapy (ART), chronic inflammation, and traditional cardiovascular risk factors (5, 6). Previous studies have demonstrated a male predominance of ACS diagnosis at an earlier age among PLWH (7). Compared to individuals without HIV, a greater prevalence of non-calcified coronary plaques has been reported in PLWH (8). Additionally, PLWH have an elevated risk of developing ACS compared with that of the general population (9). A meta-analysis found a 2-fold increased risk of CVD in HIV-infected vs. uninfected individuals (10). However, data regarding ACS characteristics in PLWH are limited, particularly in China.

Therefore, in this study, we aimed to summarize and analyze the characteristics of ACS in PLWH to further elucidate the association between HIV infection and CVD.

## Methods

### Study design and population

This study was a single-center prospective observational cohort analysis conducted at China's Shenzhen Third People's Hospital. The subjects were PLWH with acute coronary syndrome (ACS) from January 2019 to December 2023, with a total of 43 consecutive cases of HIV-positive patients with concurrent ACS collected. Concurrently, 86 non-HIV-infected individuals were selected as the control group, matched at a 1:2 ratio according to age, gender, and ACS classification criteria. All participants were aged ≥18 years and provided written informed consent. The study was approved by the institutional ethics committee (No. 2022-172) and strictly adhered to the ethical guidelines of the Helsinki Declaration.

### Control selection

HIV-uninfected controls were enrolled using a consecutive sampling framework. Specifically, for every PLWH who presented with a first episode of ACS and met the inclusion criteria, we screened the next two eligible HIV-uninfected ACS patients admitted to the same cardiac unit within ±30 days. Eligibility required that controls had no documented HIV infection (negative antibody test within 12 months), were ≥18 years old, and presented with their first ACS event. To enhance internal validity, we frequency-matched on four *a priori* confounders: age (±5 years), sex, ACS subtype (STEMI vs. NSTEMI vs. unstable angina), and calendar quarter of admission. Matching was performed at the design stage; if more than two controls met the matching criteria, the two whose age was closest to the index case were selected. We further corrected for residual imbalances (hypertension, diabetes, dyslipidemia) through multivariate logistic regression to further balance cardiovascular risk factors between the two groups.

### Data collection

Baseline demographic characteristics, cardiovascular risk factors, medical history, and laboratory results were collected through standardized case report forms and electronic medical records. Cardiovascular risk factors included current smoking, BMI, hypertension, diabetes, and dyslipidemia. Laboratory tests included D-dimer, lipid profile (TC, TG, LDL-C, HDL-C), fasting glucose, CRP, and complete blood counts including neutrophil and lymphocyte counts. Renal function indicators such as serum creatinine, serum urea nitrogen, uric acid, and estimated glomerular filtration rate (eGFR) were also recorded.

### Coronary angiography and SYNTAX scoring

To assess interobserver agreement in SYNTAX score evaluation, two experienced cardiologists independently scored all angiograms in a blinded manner. Interobserver agreement was quantified using the intraclass correlation coefficient (ICC) with a two-way random-effects model for absolute agreement [ICC(2,1)]. CAD was defined as luminal stenosis ≥50%. Lesions were classified as bifurcation, diffuse, calcified, tortuous, or thrombotic based on standard angiographic criteria. The anatomical complexity of coronary lesions was assessed using the SYNTAX score, calculated with the official online tool, as it has been validated to predict adverse outcomes in CAD.

### Definitions

ACS was diagnosed based on the Fourth Universal Definition of Myocardial Infarction and included STEMI, NSTEMI, and UA. Nonfatal MI was defined as a rise and/or fall of cardiac troponin with at least one value above the 99th percentile upper reference limit, accompanied by clinical evidence of myocardial ischemia. Cardiovascular death was defined as death resulting from an acute MI, sudden cardiac death, heart failure, or stroke. Recurrent ACS was defined as a new episode of anginal symptoms confirmed by ECG changes or elevation in cardiac biomarkers.

### Outcomes

The primary outcome was the occurrence of major adverse cardiovascular and cerebrovascular events (MACCEs) during a 2-year follow-up period. Events included cardiac death, nonfatal MI, recurrent ACS, repeat coronary revascularization, ischemic stroke, and hospitalization for heart failure or gastrointestinal bleeding. Patients underwent 24-month follow-up through regular outpatient visits, telephone interviews, and hospital record reviews after discharge. Follow-up data on MACCE events were collected at 6, 12, and 24 months post-discharge. All suspected MACCE events were independently reviewed and adjudicated by a clinical endpoint committee comprising three experienced cardiologists, who were unaware of the patients' HIV status. Adjudications were based on predefined definitions consistent with current international guidelines, with disagreements resolved through consensus.

### Statistical analysis

Continuous variables are expressed as mean of standard deviation (SD) ± or median with interquartile range (IQR), and categorical variables are expressed as counts and percentages. Student's t-*t*est or Mann–Whitney *U*-test was used for continuous variables, and chi-square test or Fisher's exact test was used for categorical variables for between-group comparisons. The factors of *P* < 0.1 in the univariate analysis were included in the multivariate regression analysis. Multivariate logistic regression was used to identify predictors of recurrent ACS and to adjust for clinically relevant variables. Odds Ratio (OR) with 95% confidence intervals (CIs) was reported. All statistical analyses were performed using SPSS version 19.0 (IBM Corp., Armonk, NY, USA) with a two-sided *P*-value of <0.05 considered statistically significant.

## Results

### Baseline characteristics

A total of 129 patients with ACS were enrolled, including 43 PLWH and 86 HIV-uninfected individuals. The majority were male (92.2%), with a mean age of 54.51 years in the PLWH group and 55.69 years in the HIV-uninfected group (*P* = 0.521). No significant differences were observed between the two groups regarding BMI, smoking status, or the prevalence of hypertension, diabetes, hyperlipidemia, or prior CVD history (all *P* > 0.05) (Table 1).

**Table 1:** Baseline demographic and angiographic characteristics.

Characteristics	HIV-infected (*N* = 43)	HIV-uninfected (*N* = 86)	*P*
Age, years	54.51 (10.06)	55.69 (9.62)	0.521
Men, *n*	40 (93.0)	79 (91.9)	1.000
Body mass index, kg/m^2^	23.88 (2.92)	24.32 (3.10)	0.439
Smoking, *n*	16 (37.2)	34 (39.5)	0.850
Coronary risk factors, *n*			
Diabetes	11 (25.6)	21 (24.4)	0.885
Hypertension	10 (23.3)	27 (31.4)	0.335
Hyperlipidemia	13 (30.2)	21 (24.4)	0.480
CVD history	7 (16.3)	16 (18.6)	0.745
Diagnosis			1.000
STEMI, *n*	11 (25.6)	22 (25.6)	
NSTEMI, *n*	13 (30.2)	26 (30.2)	
UA, *n*	19 (44.2)	38 (44.2)	
Biological parameters			
D-Dimer	0.42 (0.27)	0.70 (0.75)	0.002
Total cholesterol, mmol/L	4.48 (1.33)	4.24 (1.18)	0.296
Triglycerides, mmol/L	2.31 (1.11)	1.82 (1.62)	0.078
LDL-C, mmol/L	3.18 (1.08)	2.66 (0.92)	0.009
HDL-C, mmol/L	1.02 (0.28)	1.06 (0.25)	0.453
White blood cell count, 10^9^	7.54 (3.02)	7.87 (2.09)	0.518
LYMPH, 10^9^	1.87 (0.73)	1.63 (0.70)	0.070
NEUT, 10^9^	4.59 (2.79)	5.47 (2.60)	0.079
Serum urea	6.82 (5.73)	5.72 (3.47)	0.175
Serum creatinine	153.44 (256.52)	212.64 (975.92)	0.697
U/C	56.83 (20.46)	58.93 (27.18)	0.657
eGFR	76.45 (27.06)	81.53 (25.66)	0.304
Uric acid	393.23 (113.33)	371.02 (80.77)	0.202
CRP, mmol/L	9.10 (6.50–14.80)	5.10 (2.40–10.65)	<0.001
Number of vessels diseased			0.337
1	11 (25.6)	22 (25.6)	
2	13 (30.2)	37 (43.0)	
3	19 (44.2)	26 (30.2)	
4	0 (0)	1 (1.2)	
Culprit vessel			0.091
LAD	22 (51.2)	27 (31.4)	
LCX	7 (16.3)	14 (16.3)	
RCA	6 (14.0)	12 (14.0)	
Unknown	8 (18.6)	33 (38.4)	
Complex lesions			
Bifurcation lesions	20 (46.5)	29 (33.7)	0.158
Calcified lesions	17 (39.5)	20 (23.3)	0.054
Completely blocked	11 (25.6)	20 (23.3)	0.771
Diffuse lesions	22 (51.2)	29 (33.7)	0.085
Thrombosis lesions	11 (26.2)	24 (27.9)	0.838
SYNTAX score	21.56 (9.01)	16.77 (7.24)	0.001

STEMI, ST-segment elevation myocardial infarction; NSTEMI, non-ST-segment elevation myocardial infarction; UA, unstable angina pectoris; LDL-C, low-density lipoprotein cholesterol; HDL-C, high-density lipoprotein cholesterol; WBC, white blood cell count; LYMPH, lymphocyte count; NEUT, neutrophil count; U/C, urea/creatinine; CRP, C-reactive protein.; LAD, left anterior descending; LCX, circumflex; RCA, right coronary artery.

Laboratory analysis revealed that PLWH had significantly lower D-dimer levels (0.42 ± 0.27 vs. 0.70 ± 0.75 mg/L, *P* = 0.002), higher LDL-C levels (3.18 ± 1.08 vs. 2.66 ± 0.92 mmol/L, *P* = 0.009), and elevated CRP levels (median 9.10 vs. 5.10 mg/L, *P* < 0.001). No significant differences were noted for TC,TG, HDL-C or renal function indices (Table 1).

All patients underwent coronary angiography and guideline-directed revascularisation; none required Coronary Artery Bypass Grafting (CABG). Post-procedure, all participants received standard-of-care dual antiplatelet therapy and intensive lipid-lowering treatment, thereby removing any differential therapeutic influence on prognosis.

### Coronary angiographic features

All patients underwent coronary angiography. Although there was no significant difference in the number of diseased vessels between groups (*P* = 0.337). The mean SYNTAX score was also significantly higher in the PLWH group (21.56 ± 9.01 vs. 16.77 ± 7.24, *P* = 0.001) (Table 1). Complex lesion types showed no significant differences.

The interobserver agreement for SYNTAX scores was excellent, with an intraclass correlation coefficient [ICC(2,1)] of 0.983. The Pearson correlation coefficient between the two raters was 0.983 (*P* < 0.001), indicating a strong linear relationship.

### Two-year clinical outcomes

All participants completed the 2-year follow-up. There were two total deaths: one in the PLWH group and one in the HIV-uninfected group (2.3% vs. 1.2%, *P* = 1.000). The recurrence rate of ACS was higher in the PLWH group (18.6% vs. 7.0%, *P* = 0.089). No significant differences were found in the incidence of MI, repeat revascularization, stroke, or heart failure between the groups (Table 2).

**Table 2:** Major adverse cardiovascular events during 2-year follow-up.

Major adverse events	HIV-infected (*N* = 43)	HIV-uninfected (*N* = 86)	*P*
Death			1.000
No	42 (97.7)	85 (98.8)	
Yes	1 (2.3)	1 (1.2)	
Recurrence of ACS			0.089
No	35 (81.4)	80 (93.0)	
Yes	8 (18.6)	6 (7.0)	
Myocardial infarction			1.000
No	41 (95.3)	83 (96.5)	
Yes	2 (4.7)	3 (3.5)	
Recurrent coronary revascularization			0.415
No	38 (88.4)	81 (94.2)	
Yes	5 (11.6)	5 (5.8)	
Heart failure			0.415
No	38 (88.4)	81 (94.2)	
Yes	5 (11.6)	5 (5.8)	
Stroke			1.000
No	42 (97.7)	84 (97.7)	
Yes	1 (2.3)	2 (2.3)	

### Multivariate analysis

After screening, calcified lesions, diffuse lesions, and HIV infection were included in the multivariate analysis, with recurrent ACS and total MACCEs serving as outcomes for binary logistic regression analysis. In multivariate analysis, Calcified lesions (OR = 6.76, 95% CI: 1.86–24.54, *P* = 0.004) was significantly associated with ACS recurrence. However, after adjusting for calcified lesions, diffuse lesions and HIV infection, HIV status was not independently associated with recurrent ACS (OR = 0.58, 95% CI: 0.16–2.08, *P* = 0.405). The multivariate analysis with MACCEs showed that calcified lesions (OR = 3.97,95% CI: 1.57–10.03, *P* = 0.004) were significantly associated with MACCEs, while no link was found between HIV and MACCEs (Table 3).

**Table 3:** Multivariate logistic regression for predictors of recurrent ACS and MACCEs.

Factors	OR	95% CI	*P*
Recurrent ACS (*n* = 14):			
Calcified lesions	6.76	1.86–24.54	0.004
Diffuse lesions	3.55	0.95–13.21	0.059
HIV	0.58	0.16–2.08	0.405
MACCEs (*n* = 27):			
Calcified lesions	3.97	1.57–10.03	0.004
Diffuse lesions	2.34	0.93–5.92	0.072
HIV	0.65	0.25–1.67	0.366

### Post-hoc power calculation

This study conducted a *post-hoc* effect size analysis of the recurrence of ACS and MACCES outcomes between the HIV group and the control group under the conditions of a significance level of α = 0.05 and a power of 80%. The results showed that the actual effect size for the recurrence of ACS was 0.306, with a minimum detectable effect size of 0.246; the actual effect size for MACCES was 0.318, with a minimum detectable effect size of 0.246.

### Association between HIV-specific indicators and cardiovascular markers

Among the 43 PLWH, all patients had HIV-RNA levels < 500 copies/mL. Linear regression analysis indicated that each additional year of anti-HIV treatment was associated with a decrease of 0.12 points in SYNTAX scores (β = −0.12, 95% CI −0.23 to −0.003) and an increase of 0.01 mmol/L in LDL-C levels (β = 0.01, 95% CI −0.001 to 0.03). The CD4⁺ T-cell count (Th-Count) showed no significant association with any of the three cardiovascular markers (Table 4).

**Table 4:** Association between HIV-specific indicators and cardiovascular markers.

Variable	CRP β (95% CI)	LDL-C β (95% CI)	SYNTAX score β (95% CI)
Anti-HIV duration (per year)	−0.03 (−0.12, 0.06)	0.01 (0.001, 0.03)*	−0.12 (−0.23, −0.003)*
Th-Count (CD4⁺ cells/μL)	−0.11 (−0.29, 0.08)	0.003 (−0.03, 0.03)	−0.04 (−0.29, 0.22)

* *P* < 0.05.

## Discussion

In this single-center prospective observational study conducted in China, the characteristics of ACS in PLWH were described, analyzed in detail, and compared with those in HIV-uninfected patients with ACS. Major findings included that the SYNTAX scores were significantly higher in the PLWH group than in the HIV-uninfected group. Moreover, LDL-C and CRP levels were significantly higher in the PLWH group than in the HIV-uninfected group. In addition, the between-group recurrence risk of ACS was *P* = 0.089, falling at the margin of statistical significance. However, this non-significant difference is likely attributable to inadequate statistical power arising from the small sample size rather than a true absence of effect. Calcified lesions were significantly associated with recurrent ACS.

HIV infection and antiretroviral therapy can lead to lipid disorders (11). A previous meta-analysis showed an overall co-prevalence of dyslipidemia of 67.32% and high low-density lipoprotein (LDL-c) of 28.40% in PLWH (12). A study of PLWH receiving combination antiretroviral therapy showed a prevalence of high LDL-C in PLWH of 29.6%. High BMI is independently associated with high LDL-C (13). This study also substantiates the conclusions of previous studies that showed that patients with ACS infected with HIV exhibit higher LDL-C serum levels. Therefore, optimal control of maintaining LDL-C levels should be closely watched in the management of HIV-positive patients with ACS.

Studies have suggested that inflammation and immune deficiency related to HIV infection are risk factors for cardiovascular events in PLWH (14–16). The present study found that CRP levels in the PLWH group were significantly higher than those in the HIV-uninfected group. Abnormal immune activation associated with HIV is accompanied by a strong inflammatory response. During HIV infection and ART, inflammatory activation indicators in patients were higher than those in healthy individuals. After long-term ART, the levels of inflammatory factors may decrease; however, they cannot be completely reduced to normal levels. An increase in inflammatory activation indicators such as interleukin-6 and CRP increasesis associated with an increased mortality rate (17, 18). The pathogenesis of coronary heart disease involves pathological lipid metabolism disorders, which trigger inflammatory factors that damage the endothelium of blood vessels. The pathological changes include vascular endothelial cell injury, smooth muscle cell migration, and atherosclerosis. The nature of plaque is influenced by various factors, among which inflammatory factors play an important role. Eventually, myocardial cell ischemia and necrosis occur when the plaque becomes unstable. Among the many inflammatory factors, CRP and interleukin-6 are predictors of adverse cardiovascular events and markers of the chronic inflammatory response in atherosclerosis (19).

The current study found that the SYNTAX score of the coronary artery in the PLWH group was significantly higher than that in the HIV-uninfected group and that the incidence of coronary bifurcation lesions and vascular calcification was also significantly higher in the PLWH group than in the HIV-uninfected group. The SYNTAX score is a detailed and objective scoring system for evaluating the complexity of the coronary artery anatomy. Research has found that SYNTAX score is an independent predictor of major adverse cardiovascular events (20–22). To our knowledge, this is the second time the SYNTAX score has been used to evaluate coronary imaging features in patients with ACS and PLWH. Previously, Theodoropoulos was the first to use the SYNTAX score to quantify CAD burden. The patients had a similar extent of CAD, as measured by the presence of multivessel disease and SYNTAX score (23). Another single-center study in China used the Gensini score to measure the coronary characteristics of PLWH. The two groups had a similar extent of coronary atherosclerosis, as measured by the presence of multivessel disease and the median Gensi score (24). However, Senonera used coronary computed tomography angiography to evaluate the coronary angiography characteristics of PLWH and found that PLWH over a long term exhibited a higher severity of stenosis (25). Moreover, a meta-analysis suggested that, compared with non-HIV-infected individuals, the incidence of non-calcified CAD is higher in PLWH (8). HIV infection is a long-term, chronic inflammatory process that promotes vascular aging and calcification. This suggests that the vascular aging and calcification occur earlier in patients with HIV infection.

Current research on the intermediate prognosis of acute coronary syndrome (ACS) aligns with the findings of Matetzky's 15-month follow-up study (26) and Franck Boccara's 12-month follow-up study (27): PLWH exhibit higher ACS recurrence rates than non-HIV-positive individuals, though without statistically significant differences. Neither myocardial infarction incidence nor revascularization rate showed marked elevation. This study highlights the need to strengthen secondary prevention management for PLWH and closely monitor ACS recurrence patterns. However, the baseline angiographic characteristics of the PLWH group were more complex, and the SYNTAX score was higher, which was an independent predictor of major adverse cardiovascular events. The results of this study also found that vascular calcification is an independent predictor of stroke recurrence after 2 years. HIV infection and antiretroviral therapy associated with chronic inflammation may increase the risk of plaque rupture and atherosclerotic thrombosis (16, 28). However, traditional secondary prevention methods do not address this challenge.

The occurrence of many cardiovascular diseases, such as coronary heart disease and atrial fibrillation, is associated with HIV infection (29). Antiretroviral therapy may lead to complications such as elevated lipids, chronic inflammation, and vascular calcification. Fontas et al. demonstrated that antiretroviral treatment can cause lipid metabolism disorders, including hyperlipidemia and reduced HDL-C levels (30). A study by Rimland et al. in HIV-positive male patients showed that both protease inhibitors and non-nucleoside reverse transcriptase inhibitors elevated triglycerides (TG) and apolipoprotein CIII (apoCIII) levels (31). Robinson et al. reported increased subclinical atherosclerosis and vascular calcification in HIV patients receiving HAART treatment (32). Endothelial dysfunction and macrophage activation are two important intermediate links. Research indicates that USP18 reduces basal inflammation, senescence, and insulin resistance in coronary endothelial cells. Dolutegravir and atazanavir/r, but not maraviroc, exert opposite effects on inflammation and senescence involving USP18. In endothelial cells, dolutegravir and atazanavir/r oppositely affect pathways leading to inflammation. senescence and insulin resistance (33). HAART drugs significantly inhibit cholesterol efflux from human macrophage-derived foam cells by downregulating caveolin-1 and increasing oxidative stress, thereby suppressing cholesterol excretion and inducing endothelial dysfunction (34). These mechanisms may contribute to the development of cardiovascular diseases.

## Limitations

This study has several limitations that should be acknowledged. First, it was a single-center observational study with a relatively small sample size, particularly in the PLWH group, which may limit the statistical power and generalizability of the findings. Larger, multicenter cohorts are needed to validate our observations and improve external validity. Second, despite the analysis of key clinical variables in patients, potential residual confounders remain, particularly due to unmeasured factors such as duration of HIV infection, ART regimen, adherence, and CD4T-cell counts. These variables may affect prognostic event risk and should be included in future analyses. Third, the use of coronary angiography, while clinically standard, may underestimate the burden of subclinical or non-obstructive atherosclerosis compared to advanced imaging modalities like coronary CT or intravascular ultrasound. Incorporating multimodal imaging could enhance lesion characterization and improve mechanistic understanding. Fourth, although multivariable analyses were conducted, the low number of recurrent events which inevitably compromises the robustness and precision of the statistical adjustments. This may lead to false negative results, and marginal *P*-values may be due to insufficient statistical effect rather than a true lack of statistical association. The model diagnosis showed that the Hosmer-Lemeshow *P* values of the recurrent ACS model (14 events, 3 variables, EPV = 4.7) and the MACCE model (27events, 3 variables, EPV = 9) were 0.269 and 0.114 respectively, indicating a good fit. However, the EPV was less than 10 in both cases. Therefore, it is necessary to carry out studies with longer follow-up time and larger number of events to enhance statistical power and verify the independent predictors identified in this paper. Furthermore, given the retrospective nature of our data collection, we were unable to obtain detailed information regarding the duration of HIV infection and treatment adherence, which may have led to certain omissions. Although we observed a slight association between the duration of antiretroviral therapy and a decline in the grammar score, as well as a mild increase in low-density lipoprotein cholesterol (LDL-C) levels, the clinical significance of these associations remains unclear, constrained by the small sample size and wide confidence intervals. Future studies should prospectively collect more comprehensive HIV clinical data and validate these findings in larger sample populations. Finally, the biomarkers included in this study were limited; A broader set of inflammatory and immune activation markers may provide a deeper understanding of the biological mechanisms linking HIV to coronary artery disease.

## Conclusion

In this study, it was found that HIV-infected ACS patients had more complex coronary anatomy, higher CRP levels with LDL-C levels, and higher ACS recurrence rates during the 2-year follow-up period. These findings highlight the unique cardiovascular phenotype of individuals living with HIV, characterized by increased inflammation and metabolic disorders. For patients, the results highlight the importance of enhanced long-term cardiovascular monitoring and the need for targeted secondary prevention strategies beyond standard lipid-lowering treatments. For clinicians, our findings suggest that greater attention should be given to coronary lesion complexity and metabolic control in PLWH with ACS, and that management should be tailored to address their unique risk profiles. From a public health perspective, this study emphasizes the growing burden of CVD in aging PLWH and calls for integrated care models that bridge infectious disease management with cardiovascular prevention. Collectively, our findings support the need for future multicenter studies with larger cohorts and comprehensive biomarker profiling to further elucidate the mechanisms of cardiovascular risk in HIV, and to inform evidence-based strategies that reduce recurrence and improve long-term outcomes in this vulnerable population.

## Data Availability

The raw data supporting the conclusions of this article will be made available by the authors, without undue reservation.
